# Epidemiologic Features of Four Successive Annual Outbreaks of Bubonic Plague in Mahajanga, Madagascar

**DOI:** 10.3201/eid0803.010250

**Published:** 2002-03

**Authors:** Pascal Boisier, Lila Rahalison, Monique Rasolomaharo, Maherisoa Ratsitorahina, Mahafaly Mahafaly, Maminirana Razafimahefa, Jean-Marc Duplantier, Lala Ratsifasoamanana, Suzanne Chanteau

**Affiliations:** * Institut Pasteur, World Health Organization Collaborating Center for Plague, Antananarivo, Madagascar; † Hopital d'Instruction des Armees Robert Picque, Bordeaux, France; ‡ Androva Hospital, Mahajanga, Madagascar; § Institut de Recherches pour le Developpement, Antananarivo, Madagascar; ¶ Plague National Control Programme, Ministry of Health, Antananarivo, Madagascar

**Keywords:** Plague, Yersinia pestis, Madagascar, urban outbreak, epidemiology

## Abstract

From 1995 to 1998, outbreaks of bubonic plague occurred annually in the coastal city of Mahajanga, Madagascar. A total of 1,702 clinically suspected cases of bubonic plague were reported, including 515 laboratory confirmed by *Yersinia pestis* isolation (297), enzyme-linked immunosorbent assay, or both. Incidence was higher in males and young persons. Most buboes were inguinal, but children had a higher frequency of cervical or axillary buboes. Among laboratory-confirmed hospitalized patients, the case-fatality rate was 7.9%, although all *Y. pestis* isolates were sensitive to streptomycin, the recommended antibiotic. In this tropical city, plague outbreaks occur during the dry and cool season. Most cases are concentrated in the same crowded and insanitary districts, a result of close contact among humans, rats, and shrews. Plague remains an important public health problem in Madagascar, and the potential is substantial for spread to other coastal cities and abroad.

Plague is enzootic in the central highlands of Madagascar, where approximately 200 to 400 bacteriologically confirmed or presumptive cases are reported each year to the World Health Organization; 1,500 to 2,500 clinically suspected cases are reported by the national surveillance system (*1*,*2*). In this island, the main reservoir of *Y. pestis* is the black rat (*Rattus rattus*) and the main vector the rat flea (*Xenopsylla cheopis)* (*3*). From the arrival of plague in Madagascar in 1898 until the 1920s, plague occurred in several harbors around the island. It disappeared progressively from the coastal areas as soon as it reached the central highlands where, above an altitude of about 800 m, it found a suitable environment. A period of quiescence in the coastal areas lasted more than 60 years. Then, in August 1991, a sudden outbreak lasting 7 months occurred in the harbor of Mahajanga (*4*). During the next 3 years, when neither an epizootic nor a human case was reported, the outbreak was considered an isolated epidemiologic incident. However, in 1995 a new epidemic occurred (*5*,*6*), which was followed by three others, in 1996, 1997, and 1998. These outbreaks accounted for approximately 30% of the reported cases of plague in Madagascar during the period. We describe the main epidemiologic features of these four urban outbreaks in this exceptional resurgent coastal plague focus.

## <H1> Population and Methods

In Madagascar, health workers must report all clinically suspected cases of plague to the national surveillance system. For each patient, a biological sample (bubo aspirate, sputum, or postmortem liver or lung puncture, whenever appropriate) has to be collected and sent by mail to the National Reference Laboratory at the Institut Pasteur de Madagascar in the capital, Antananarivo. The delivery time is frequently 2-3 weeks after the specimen was collected, and the only reliable method to allow bacteriologic diagnosis remains bubo aspiration (as well as sputum and postmortem liver or lung puncture, whenever appropriate) and transportation on a swab in Cary-Blair medium. Blood samples for culture were not adopted in Madagascar since the likelihood of isolating *Y. pestis* is approximately twice as high in buboes as in blood. In August and September 1997, a temporary bacteriology laboratory was established in Mahajanga; thus, all the biological samples collected during these 2 months were processed on site.

The confirmatory diagnosis was based on bacteriologic methods. A case of plague was considered to be confirmed as soon as a strain of *Y pestis* was isolated by culture or mouse inoculation. A patient was considered to have a presumptive plague case when *Y. pestis* could not be isolated but gram-negative bacillus, with morphologic patterns of *Y. pesti*, could be detected on smear. Microscopy lacks both sensitivity and specificity, and the isolation of *Y. pestis* requires at least 6 days. Prior treatment of patients with antibiotics impedes the culture and may lead to false-negative results. Physicians were asked to collect an acute-phase serum sample before treatment and a convalescent-phase serum sample at least 7 days after the onset of disease. Whenever available, the sera and bubo aspirates were tested for F1-antigen by immunocapture enzyme-linked immunosorbent assay (ELISA) (*7*,*8*) and for anti-F1 antibodies by the classical indirect ELISA method (*9*).

In this study, the bacteriologically confirmed or presumptive patients, the ELISA-positive patients (F1 antigen or antibodies), or both were defined as having laboratory-confirmed cases. Clinical and epidemiologic data were collected on standard forms, after the patients or their families were interviewed. Reporting to the national surveillance system was assumed to be thorough, as confirmed by a seroepidemiologic survey in 1999 (*10*). In Madagascar all *Y. pestis* isolates are screened for their in-vitro resistance to streptomycin, gentamicin, chloramphenicol, tetracycline, sulfamethoxazole-trimethoprim, and ampicillin.

The geographic distribution of plague patients was visualized by using a simplified map of Mahajanga. For this purpose, the city was divided into four areas. We defined the boundaries of these areas by aggregating districts that were comparable for population density, sanitation level, and housing type.

## <H1> Results

In 1995, the first identified case occurred in March, followed by several sporadic cases in May and July. The outbreak proper started by mid-August. From 1995 to 1998, 1,702 clinically suspected bubonic plague cases were reported; 335 were considered confirmed (297) or presumptive (38) cases after bacteriologic testing. None of the *Y. pestis* isolates was recovered from sputum. When either F1 antigen capture or anti-F1 ELISA assays were used, 180 more cases were laboratory confirmed. In all, 515 persons were considered to have contracted plague from January 1, 1995, to December 31, 1998.

For each of the 4 years we studied, a biological result was available for 88.5%, 98.7%, 97.2%, and 99.5% of the suspected patients, respectively. When bacteriologic methods were used, the annual confirmation rates were 22.2%, 14.8%, 30.1%, and 30.3%, respectively. The proportions represented by bacteriologically confirmed cases among the total number of laboratory-confirmed patients were 72.3%, 83.6%, 98.7%, and 88.1%, respectively. Detailed laboratory results are summarized in the Table.

**Table:** Results of bacteriology testing for *Yersinia pestis*, Mahajanga, Madagascar, by year

	1995	1996	1997	1998	Total
Total suspected cases	558	399	539	206	1,702
Laboratory-confirmed^a^ cases	117	97	214	87	515
Bacteriology					
Number tested	342	330	501	195	1,368
Number confirmed	55	41	149	52	297
Number presumptive	21	8	2	7	38
Number positive^b^	76	49	151	59	335
Percent positive	22.2	14.8	30.1	30.3	24.5
F1 antigen Elisa					
Number tested	433	335	413	189	1,370
Number positive	38	25	131	35	229
Percent positive	8.8	7.5	31.7	18.5	16.7
Anti-F1 antibodies Elisa					
Number tested	365	344	396	191	1,296
Number positive	68	59	137	48	312
Percent positive	18.6	17.2	34.6	25.1	24.1

^a^Confirmed + presumptive and/or enzyme-linked immunosorbent-assay (Elisa) positive. ^b^Confirmed + presumptive cases.

Two of the 297 *Y. pestis* isolates from Mahajanga patients were resistant to one of the tested antibiotics, one to chloramphenicol in 1996 and one to ampicillin in 1998.

**Figure 1:**
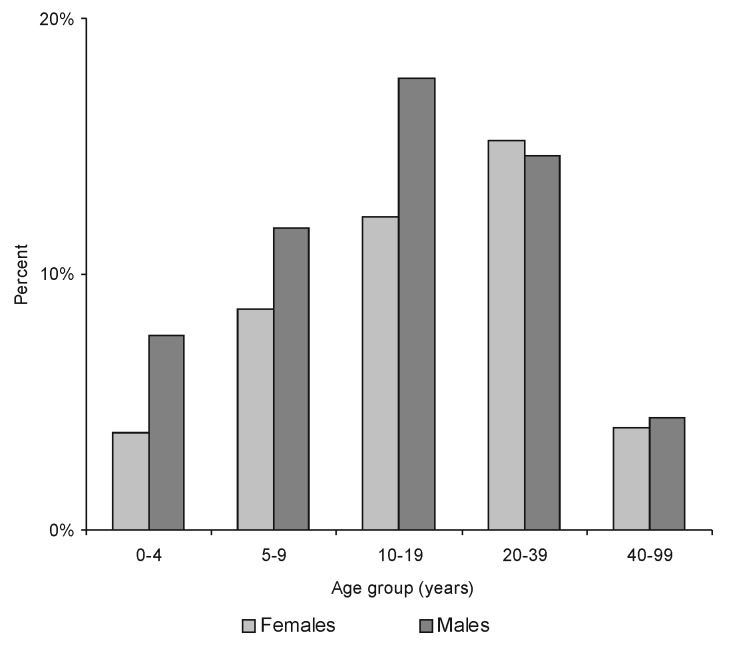
Age and sex-distribution of laboratory-confirmed bubonic plague cases, Mahajanga, Madagascar.

The proportion of males (56.1%) was significantly higher among cases than in the general population (p=0.006). The age and sex-distribution of patients with laboratory-confirmed cases remained unchanged during the 4 years (Figure 1). The median age of patients was 15 years, and 75% of patients were <25 years old. Although the highest incidence of the disease was observed in 5-to 19-year-old persons, 59 cases occurred in children <5 years old; 2 were <1 year old.

**Figure 2:**
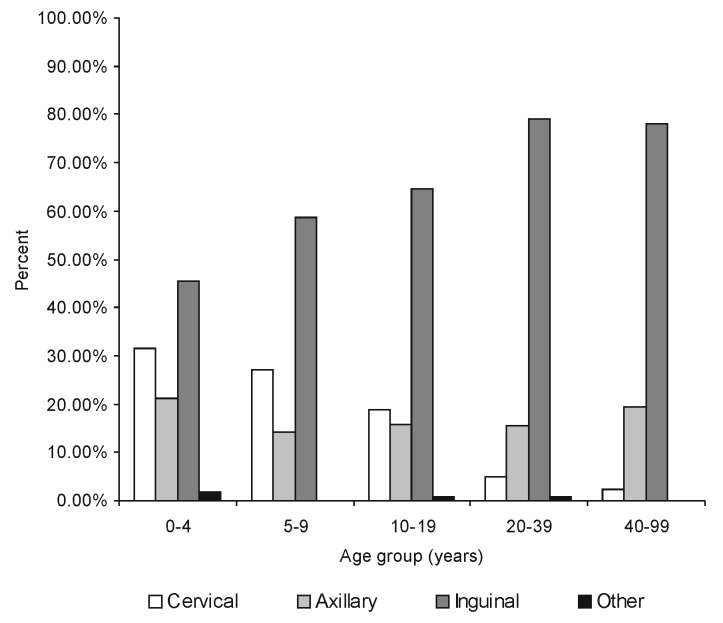
Distribution of bubo location according to age in laboratory-confirmed bubonic plague cases, Mahajanga, Madagascar.

Among laboratory-confirmed cases, a significantly higher frequency of cervical and axillary buboes occurred in children; by contrast, inguinal buboes represented about 80% of cases in persons ≥20 years of age (p<10^-7^). The distribution of bubo location according to age is shown in Figure 2. Body temperatures were available for 454 of persons with laboratory-confirmed cases: the median temperature was 39.5°C (25th and 75th percentiles were 38.2°C and 40°C). Diarrhea (7.1%), prostration (4.5%), and coma (1%) were the other most frequently reported symptoms.

A total of 507 laboratory-confirmed patients were admitted to hospital; 40 (7.9%) of them died. The case-fatality rate was not significantly different by year (7.1%, 9.3%, 6.7%, and 10.3% in 1995, 1996, 1997, and 1998, respectively). Nor was this rate related to age, sex, bubo location, or delay between onset of disease (as reported by the patients) and initiation of treatment. Lethality was also not correlated with drug susceptibility of *Y. pestis* isolates, since they were all sensitive to streptomycin, the drug recommended by the national program. Only the body temperature at admission to the hospital was significantly higher in the group of deceased patients than in recovered (39.6°C vs. 39.1°C, p=0.01).

**Figure 3:**
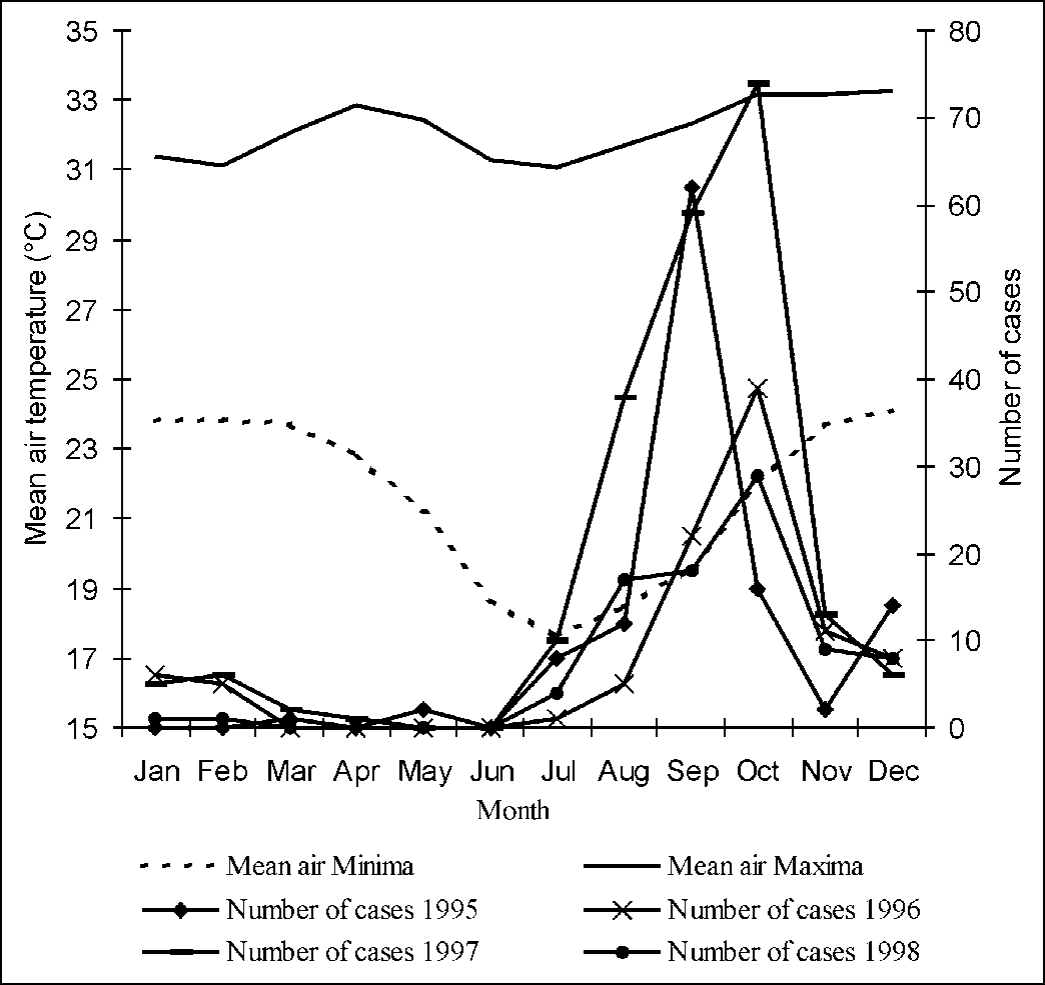
Mean air temperatures and month-distribution of laboratory-confirmed cases of bubonic plague, Mahajanga, Madagascar, since 1995.

Most (76%) patients were reported during August through October during the dry season, a peak that occurs every year. The temperature patterns in Mahajanga and the monthly distribution of laboratory-confirmed cases are related. The outbreaks used to occur in July, when the lowest temperature is the lowest of the year, and ceased in November, when the temperature rebounds (Figure 3).

Among the 357 laboratory-confirmed plague patients for whom data were available about rat deaths within the 15 days preceding onset of disease, 203 (56.9%) had found dead rats indoors or in the vicinity of their homes; 154 (43.1%) had not noticed dead rats in their surroundings. Of the 203 who had, 117 (57.6%) had found the dead rats inside their homes. The confirmation rate was higher among persons who reported rat deaths in their surroundings than among persons not reporting such deaths **(**57.7% versus 24.6%, p<10^-7^).

**Figure 4:**
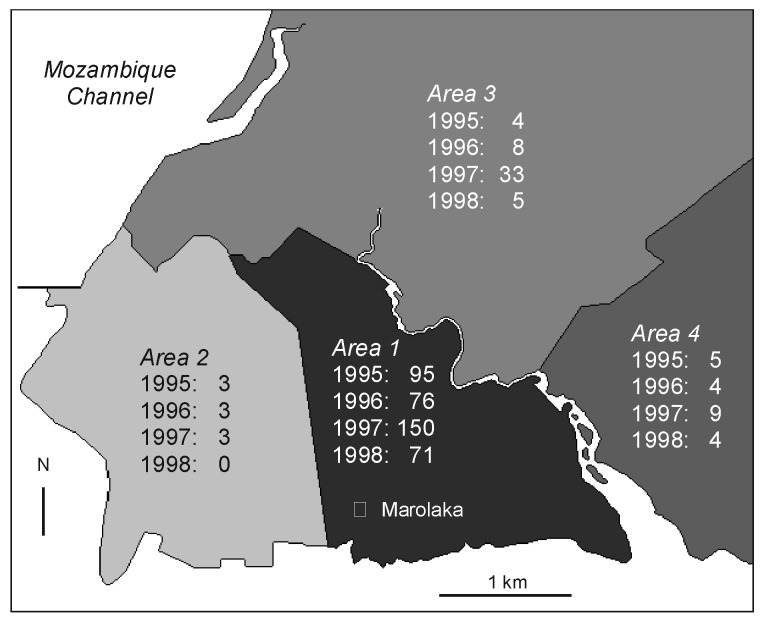
Incidence of laboratory-confirmed bubonic plague cases according to the patients’ place of residence, in Mahajanga, Madagascar.

The geographic distribution of plague laboratory-confirmed cases according to districts is shown in Figure 4. The residence of patients could be clearly identified on this map for 473 (91.8%) of the laboratory-confirmed cases. The incidence of plague differed sharply according to districts: most patients (82.9%) lived in Area 1, which pools the most unhealthy and densely populated districts of the town. The southwestern part of the city (Area 2), including the harbor and the old colonial town, had few cases and did not show any trend towards increasing incidence. Areas 3 and 4, greener and less populous suburbs of Mahajanga, showed an intermediate incidence. The increase in cases in 1997, especially in Area 3, was no longer occurring in 1998.

## <H1> Discussion

World plague foci are mostly restricted to temperate climate highlands such as regions in East Africa, central Asia, and the American Southwest; outbreaks in coastal areas have become rare. Thus, the situation in the harbor of Mahajanga, where plague has found favorable conditions and seems to have established itself, is noteworthy. During 4 successive years, 97 to 214 laboratory-confirmed plague cases were reported annually, raising questions about epidemiologic determinants of this disease’s having taken roots in this tropical city. Before its sudden reemergence in 1991 after more than 60 years of quiescence, plague in Madagascar was supposedly restricted to areas above 800 m because of climatic constraints that influence the proliferation of fleas and *Y. pestis* (*11*). In Mahajanga, as had already been observed in 1907 (*12*), the plague season starts in July or August, during the dry season, when the air temperature is the lowest. This is in contrast with the central highlands, where most of the cases occur between October and February, during the warmer rainy season (*2*). Indeed, during the plague season for both the coastal and plateau regions, the minimum temperature is about the same, between 17° and 22°C. Recent studies have shown that the plague season in the central highlands is clearly linked to the abundance both of fleas and the black rat*, R. rattus*, the main reservoir and virtually the only small mammal found in houses (95% of captures in traps) (*13*). In Mahajanga, ongoing studies have shown that the Asiatic shrew, *Suncus murinus,* accounts for up to 75 % of the trapped animals and is a regular carrier of the rat flea, *X. cheopis* (Duplantier et al., unpub. data). Moreover, the seroprevalence among shrews trapped during the postepidemic period was 43% in 1995. *Y. pestis* strains were also isolated from five shrews in 1996 and 1997 (Chanteau et al., unpub. data ). All these findings strongly suggest the determinant role of shrews as a previously unrecognized host of *Y. pestis* in the epidemiologic cycle of plague in Mahajanga. This new parameter complicates and revises the classical rodent-flea-human triad. In Southeast Asia, the role of *S. murinus* in plague is established (*14*,*15*).

The epidemic wave of plague that started in August 1991 and lasted until February 1992 was confined to the neighborhood surrounding the main market in Area 1; this region, and more precisely a place named Marolaka, was considered to be the epicenter of the outbreak (*4*). The source of the initial contamination was probably inland, due to the trading of agricultural products from the northern plague foci to the marketplace, as suggested by results of the *Y. pestis* genotyping by pulsed-field gel electrophoresis (Buchrieser et al., manuscript in preparation). Although we think that an inland reintroduction of the infection is unlikely to occur for 4 consecutive years under the same pattern, we cannot exclude this hypothesis until molecular analysis of the isolates is available. The epidemic ring did not extend to the other areas of the town, and this quiescence lasted 3 years, during which information about hosts and fleas was scarce. The starting point for the second epidemic wave described here was in exactly the same zone of the marketplace; however, this time, the plague front extended to the other three areas. Over the 4 years, the incidence was higher in Area 1 than in the others, although in 1997 Area 3 was clearly affected. The plague front did not extend further than 10 to 15 km from town.

A geomedical survey (Rakotoarisoa S, unpub. data) concluded that three different types of structures were present in the entire city of Mahajanga**.** Area 2 is almost comparable with a European city with its wide, paved streets, sewer network, store buildings with apartments in the upper floors, and low population density); Areas 3 and 4 are semirural suburbs with low population density. In contrast, Area 1, which was the epicenter for the two waves of the outbreak, is densely populated with very poor people. This area also includes the two largest markets, which generate the town’s largest amount of rubbish. Therefore, while no physical barrier separates Area 1 from Area 2, the lower incidence in the latter could be related to the slimmer chance of contact between humans and reservoirs of plague, in short, to better housing conditions.

Clinically, despite some published claims that the clinical diagnosis of bubonic plague is straightforward**,** field data show a bacteriologic confirmation rate no greater than 30% for the best years. This rate improved in 1997 and 1998, compared with 1995 and 1996, suggesting that physicians are becoming more skilled at making this diagnosis. The increased confirmation rate was also due to their increased skill in collecting bubo pus and the progressive shortening of the delay before the samples were analyzed in a laboratory. Except during the 2 months of August and September 1997, bubo samples arrived at the central plague laboratory in Antananarivo as long as 2 or 3 weeks after being collected, which led to false-negative results because they were contaminated or the plague bacillus had died. The use of ELISA methods to detect either F1 antigen in acute-phase bubo samples or antibodies in convalescent-phase serum contributed to the confirmation of 35% of the total laboratory-confirmed cases. However, bacteriology and ELISAs are used as retrospective tools to confirm plague. Only a rapid diagnostic test such as the F1 dipstick assay is a valuable tool for health workers (*8*).

The higher incidence of bubonic plague in males than females and in young persons rather than in adults is a constant epidemiologic feature in Madagascar, whether in Mahajanga or the highlands (*1*–*3*). In published studies, gender differences in incidence rates differ by country (*16*). In India, females were more frequently infected; in the city of Hai-nan, China, males and females were equally affected; and in Manchuria, the situation was similar to that in Madagascar (*16*)**.** Despite its being well accepted that incidence is linked to extrinsic more than intrinsic factors, we could not find any link to occupational behavior.

The effects of human age on the relationship between rat fleas and people and of gender on this disease deserve further study. The observation of a relationship between the age and the bubo location is common in Madagascar, although it has not been described elsewhere. As it is widely accepted that the location of the bubo is dependent on the place where the injection of *Y. pestis* occurred**,** we infer that infective flea bites more often involve the upper extremities in children than in adults. In the urban plague focus of Mahajanga, as in the central highlands, transmission is believed to occur mostly inside houses. Although we do not have any indications that children have specific risks, such as handling dead rodents in play, children do spend more time close to the floor (e.g., games, sleeping) than adults) and therefore are closer to fleas.

From the start, the absence of pneumonic plague has been remarkable. The natural course of bubonic plague can lead to secondary pneumonic plague, which can give rise to highly contagious cases of primary pneumonic plague in contacts, as seen every year in the highlands of Madagascar (*17*). Yet not a single contact case has been reported, even though several bubonic patients died before being diagnosed and treated and thus contacts remained unidentified who could have benefited from chemoprophylaxis. This observation fits with early studies describing peumonic plague only in temperate places. As far back as 1929, Thiroux pointed out that pneumonic plague outbreaks had never been observed in Madagascar in areas where the absolute minimum temperatures did not remain regularly under 16°C for several consecutive days (*18*). As early as 1907, in the absence of effective treatment and chemoprophylaxis, only four cases of pneumonic plague had been observed among 72 plague cases during the first epidemic in Mahajanga. The absence of lung infection apparently is not related to a particular strain of the plague bacillus since the *Y. pestis* strain that currently circulates in Mahajanga was likely introduced from the highlands in 1991, as demonstrated by pulsed-field gel electrophoresis genotyping (Buchrieser et al, manuscript in preparation).

The case-fatality rate is somewhat higher than reported in published studies, and it does not show any trend towards decreasing. However, we considered only laboratory-confirmed cases, and technical conditions in Mahajanga hospital are poor. We believe that a major proportion of treatment failures could be avoided if patients did not wait 2 days or more before coming to hospital. Moreover, dates of onset seem to be questionable for some serious cases; apparently families are often reluctant to imply that they have been negligent in managing the patient at home, or they have resorted to traditional healing before referring the patient to a hospital. Up to now, antibiotics used to treat the disease (streptomycin is the recommended treatment in Madagascar) have been totally effective against *Y. pestis* strains from Mahajanga. The discovery of one chloramphenicol-resistant isolate and one ampicillin-resistant isolate requires that the country maintain an efficient bacteriology surveillance system. The appearance and spread of multiresistant *Y. pestis* strains, such as the two isolated in 1995 in the highlands, are cause for concern (*19*,*20*).

Plague is still a threat in Madagascar and is no longer restricted to areas in the highlands over 800 m. A bubonic plague outbreak has been reported recently at 500 m altitude (*21*,*22*). Such epidemics as we describe may occur in any other coastal city where the shrew *S. murinus* and the flea *X. cheopis* are present. Because trade between the highlands and the ports has intensified, an active program for surveillance and monitoring of plague borders must be maintained. Improved ways to rat-proof structures should also be encouraged.
